# Advances in 3D Printing for Removable Prosthetics—Insights and Perspectives

**DOI:** 10.1111/jerd.70097

**Published:** 2026-01-06

**Authors:** O. Schubert, D. Edelhoff, J. F. Güth, J. Schweiger

**Affiliations:** ^1^ Department of Prosthetic Dentistry University Hospital, LMU Munich Munich Germany

**Keywords:** 3D printing, additive manufacturing, complete dentures, digital dentistry, implant overdentures, occlusal splints, removable partial dentures

## Abstract

**Objective:**

This review summarizes recent advances in three‐dimensional (3D) printing for removable prosthodontics, including complete dentures, removable partial dentures, implant‐retained overdentures, and occlusal splints. The aim is to provide clinicians with an overview of current technologies, clinical performance, and future perspectives.

**Overview:**

While subtractive methods are well established, additive manufacturing has recently gained exponential importance, especially in removable restorations. Vat‐photopolymerization resins are widely applied for denture bases, teeth, and occlusal splints, while laser sintering enables fabrication of metal frameworks and implant attachments with high accuracy. Clinical studies indicate that printed complete dentures achieve outcomes in adaptation and patient satisfaction comparable to conventional or milled prostheses, with reduced appointment numbers and digital reproducibility. Removable partial denture frameworks and implant‐overdenture attachments fabricated by selective laser melting demonstrate promising accuracy and mechanical properties, though process variability and post‐processing remain critical aspects. Printed occlusal splints are widely adopted and offer adequate fit and comfort, though material‐related challenges and limited long‐term data are concerns.

**Conclusions:**

3D printing emerges as a reliable option for removable dentures, frameworks, and occlusal devices. Future developments in multi‐material printing, durable and bioactive resins, and standardized clinical protocols will further define its role in prosthodontics.

**Clinical Significance:**

3D printing is transforming removable prosthodontics. Clinicians can already benefit from streamlined workflows, a standardized process chain, reduced treatment time, and the ability to reproduce dentures, while patients gain from improved comfort and fewer visits. Awareness of material limitations and processing‐related specifics remains essential for long‐term success. Looking ahead, multi‐material printing—allowing simultaneous fabrication of denture bases, teeth, and maybe frameworks—holds promise for improving structural integrity, esthetics, and overall clinical efficiency.

## 1 Introduction

Digital dental technologies have undergone rapid innovation and market adoption in recent years. While subtractive CAD/CAM (Computer‐Aided Design/Computer‐Aided Manufacturing) remains a mature and clinically well‐established approach, additive manufacturing (AM), commonly referred to as 3D printing, has gained substantial relevance and expanded its clinical scope [1, 2].

The dental digital workflow comprises three core processes regardless of the manufacturing method: (1) acquisition of surface data via intraoral or model scanning, (2) data processing (CAD), and (3) production of the physical restoration [3]. Transfer of CAD data into a physical device can proceed additively (i.e., 3D printing) or subtractively, by milling [4].

Intraoral scanning (IOS) has advanced considerably, offering efficient data capture, real‐time feedback, patient comfort, and accuracy comparable to conventional impressions [5, 6]. Modern IOS devices also integrate diagnostic and treatment‐planning tools such as caries detection, tooth wear monitoring, and AI‐driven segmentation [6]. Open system architecture and compatibility with various CAD/CAM software platforms further facilitate seamless integration into digital workflows.

**FIGURE 1:**
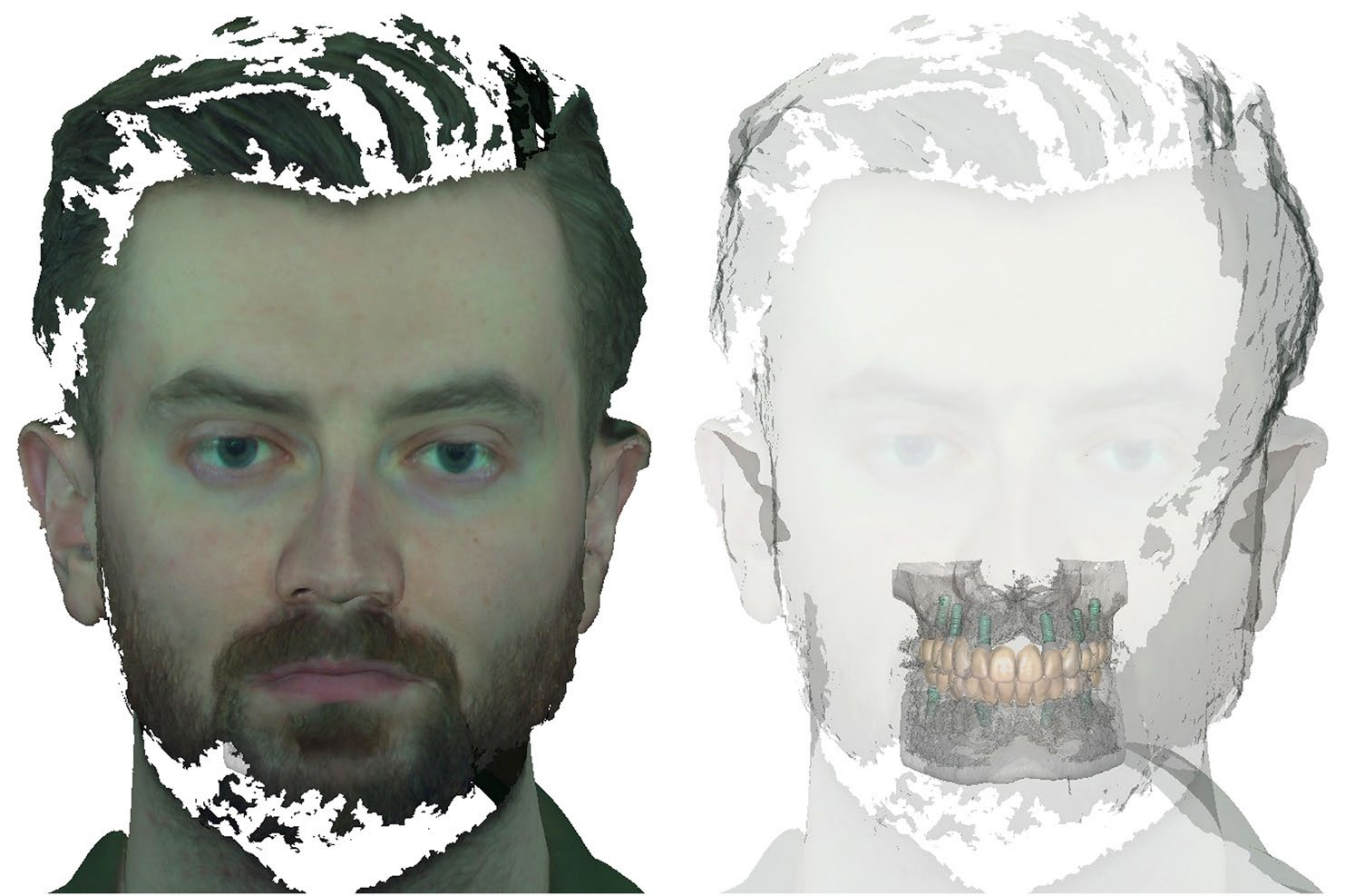
A digital twin of the patient comprising intraoral surface data, radiographic data, and a face scan, enabling comprehensive prosthodontic and implant pre‐planning (DentalCAD, 3.2 Elefsina; Exocad, Darmstadt, Germany), including functional and esthetic aspects, in a patient with early tooth loss due to a rare genetic disease (Informed consent was obtained from the patient).

CAD software has likewise progressed, with more intuitive user interfaces, improved efficiency, and increasing incorporation of AI. Partial integration into cloud‐based platforms now allows fusion of IOS with jaw movement records, facial scans, 3D radiographic imaging, and photographs. This supports creation of a digital “avatar” or “twin” of the patient—a concept already explored in medicine (Figure 1) [7]. Software also provides a wider range of restorative options, including complex multi‐material and multi‐layer structures [8, 9], thereby creating new clinical indications and demand for 3D printing.

Vat‐polymerization dominates dental 3D printing, including projector‐based stereolithography (DLP), laser‐based stereolithography (SLA), and, more recently, mask‐based stereolithography (mSLA/LCD). Material jetting, similar to 2D‐printing, is another option [2, 10, 11]. For metals, selective laser melting (SLM), also known as direct metal laser sintering (DMLS), is most common [11, 12].

Currently available printable materials can be grouped into polymers, metals, and ceramics. Light‐curing resins and PMMA dominate clinical use for dentures, splints, and surgical guides, although their mechanical properties remain inferior to milled materials and long‐term biocompatibility data are still limited. By contrast, cobalt–chromium and titanium alloys produced by SLM are well established for frameworks and bars, providing high accuracy and strength [11, 13, 14].

**FIGURE 2:**
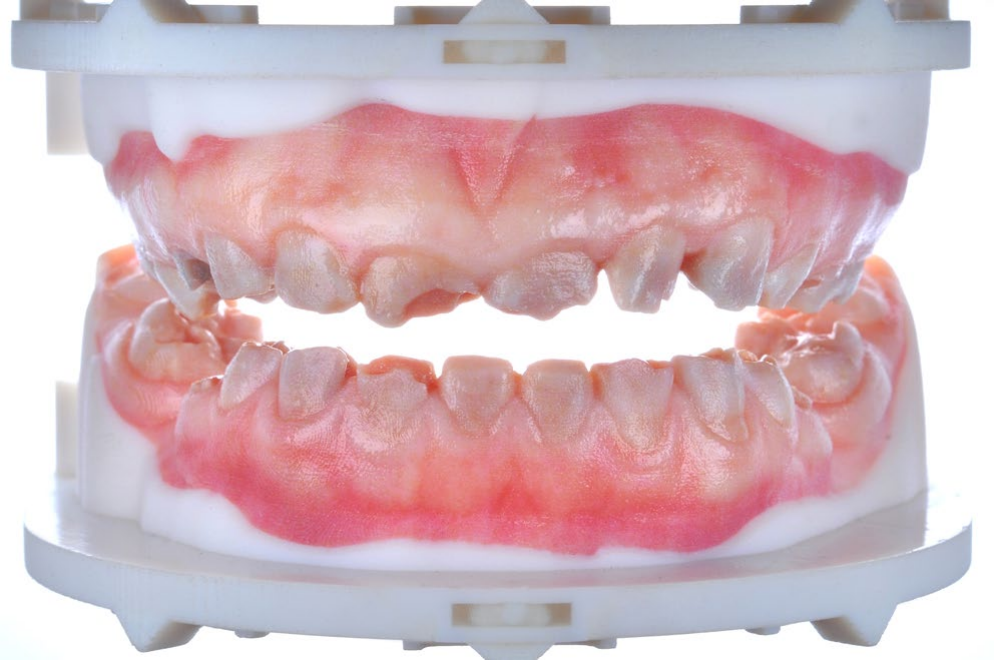
Printed model in lifelike colors (3D Medical Print, Lenzing, Austria), displaying dentition affected by dentinogenesis imperfecta.

Compared with subtractive CAD/CAM, 3D printing offers several advantages. In milling, material properties are predetermined in the prefabricated blank, whereas 3D printing allows modification of characteristics such as color or mechanical properties during the build. Multi‐material printing already enables lifelike reproduction of oral situations (Figure 2). Furthermore, depending on the method, many restorations can be fabricated within a short time and with efficient use of resources. In this context, 3D printing is particularly advantageous for large‐volume components with complex surface geometries, that is, removable dentures and fixed full‐arch restorations.

Subtractive technology remains valuable due to its well‐documented production methods, high accuracy, and stable material properties. Extensive long‐term clinical data also exist. However, it involves high investment costs, significant material waste, tool wear, and geometric limitations imposed by milling tools [15, 16].

For a long time, AM in dentistry was limited to models, surgical guides, and impression trays. Today, however, its scope has expanded not only to fixed prosthodontics but also to complete dentures, removable partial dentures, implant‐retained overdentures, and occlusal splints.

This narrative review is based on a targeted literature search in PubMed and Scopus focusing on 3D printing in removable prosthodontics. Priority was given to clinical studies, systematic reviews, and relevant in vitro investigations addressing complete dentures, removable partial dentures, implant‐retained overdentures, and occlusal splints, with emphasis on clinically established workflows and emerging technologies.

## 2 Indications for 3D Printing in Removable Prosthodontics

### 2.1 3D Printing in Complete Dentures (CD)

3D printing of complete dentures has evolved from trial and duplicate prostheses to definitive dentures, mainly using vat‐photopolymerization but also material jetting. Clinical studies report acceptable fit, adaptation, and patient satisfaction, with outcomes comparable to conventional or milled dentures [17]. Systematic reviews confirm reduced appointments and chairside time, though esthetic and mechanical limitations remain [18].

**FIGURE 3:**
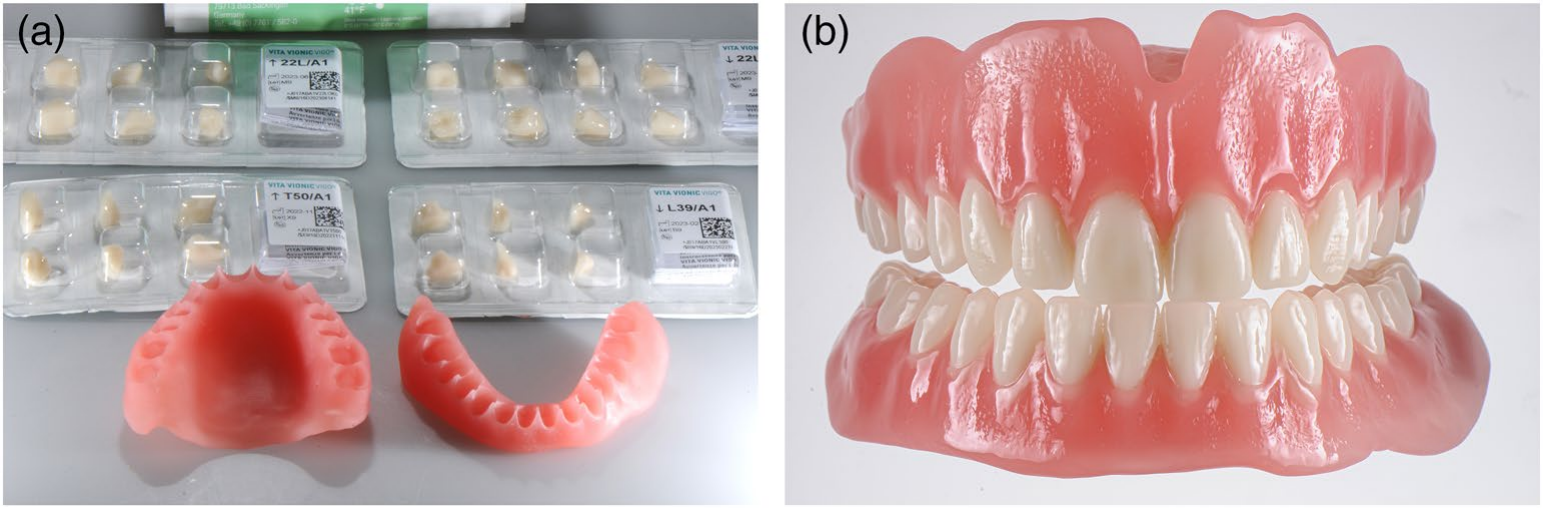
(a) Complete denture assembly in a multi‐part approach combining a printed base (Printodent GR‐14.2 Denture HI; Pro3dure, Iserlohn, Germany) with prefabricated teeth (Vita Vionic Vigo; VITA Zahnfabrik, Bad Säckingen, Germany). (b) Final denture after bonding of base and teeth and finalization (Printodent GR‐14.2 Denture HI; Pro3dure; Vita Vionic Vigo; VITA Zahnfabrik).

**FIGURE 4:**
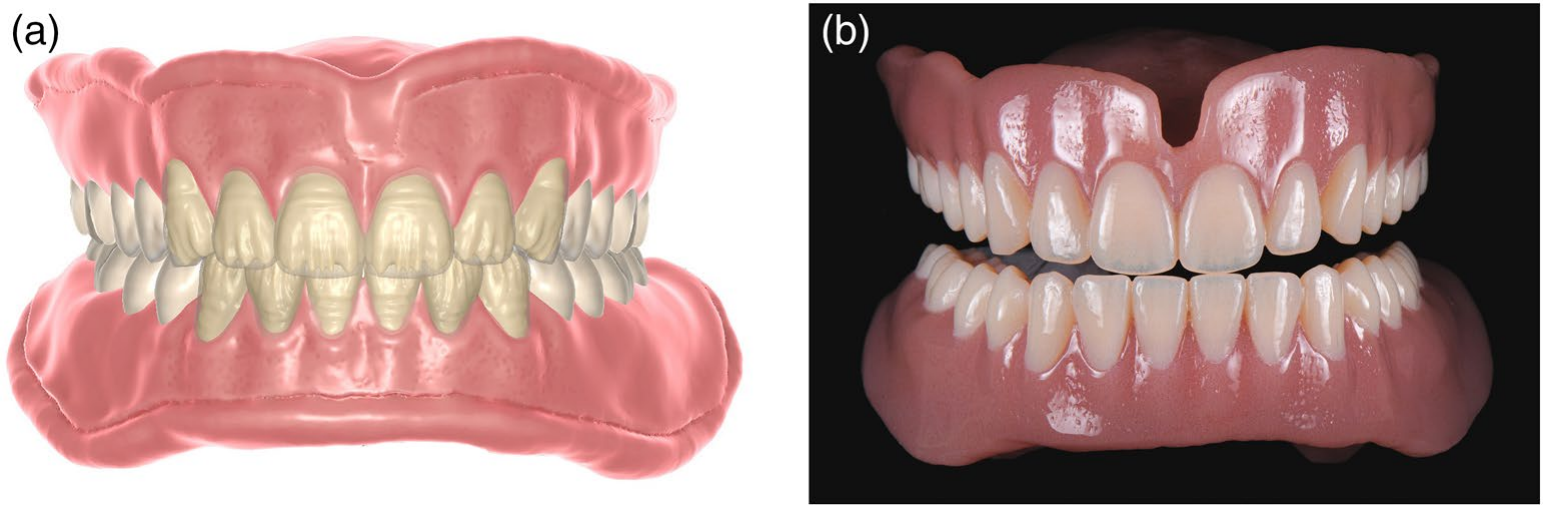
(a) CAD (DentalCAD; Exocad) of a complete denture in a multi‐material, multi‐layer single build, enabling efficiency, stability, and a highly esthetic appearance. (b) Complete denture fabricated in a multi‐material, multi‐layer approach (TrueDent Denture; Stratasys, Rehovot, Israel), printed on a J5 DentaJet PolyJet printer (Stratasys).

**FIGURE 5:**
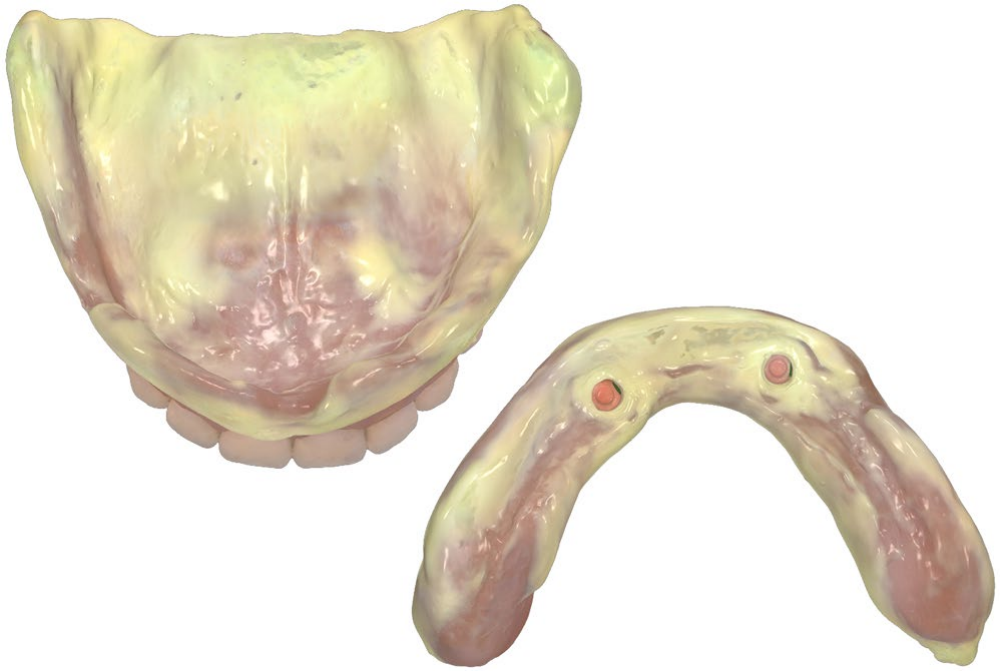
IOS data (TRIOS 4; 3Shape, Copenhagen, Denmark) of relined dentures in a case of residual ridge resorption. The data can be used to manufacture a copy denture with the necessary modifications.

The three main approaches in 3D‐printed dentures are monolithic printing of base and teeth in a single process and printing with separate bonding of base and tooth arch or single teeth, with prefabricated or printed teeth (Figure 3a,b). Recently, multi‐material printing, allowing for simultaneous printing of bases and teeth in a monolithic design, is gaining attraction (Figure 4) [19, 20]. The combination of bases and teeth within a multi‐material and multilayer single build appears to be a very promising approach (Figure 4a,b). Especially intriguing is the application of 3D‐printed duplicate, or “copy,” dentures in cases of loss, damage, or wear, as well as when modifications such as relining after residual ridge resorption are required (Figure 5).

### 2.2 3D‐Printing in Removable Partial Dentures (RPDs)

3D printing of removable partial denture frameworks is mainly performed with selective laser melting (SLM) or direct metal laser sintering (DMLS) of cobalt–chromium or titanium alloys [12, 21, 22]. These methods eliminate wax‐up and casting, enabling reproducible, model‐free fabrication. In vitro and clinical studies report clinically acceptable fit, precision, and retention, often with less variability than conventional casting [23, 24, 25].

**FIGURE 6:**
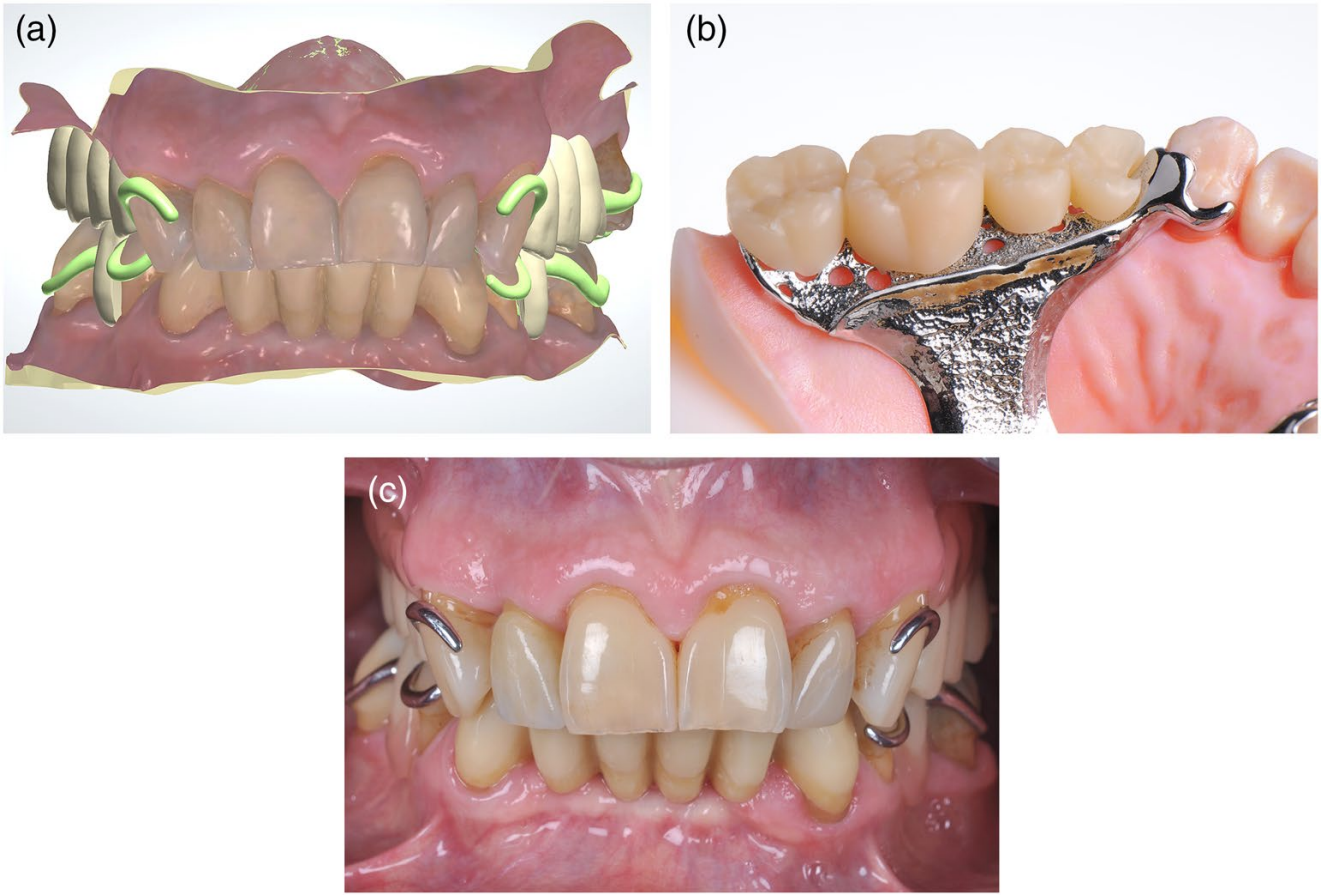
(a) CAD (Dental Designer RPD; 3Shape) of a removable partial denture (RPD) including framework and teeth. (b) Framework (WIRONIUM RP polished; BEGO, Bremen, Germany) and teeth (VarseoSmile TriniQ; BEGO) on a colored model (3D Medical Print), all 3D‐printed—before assembly. (c) Clinical impression of the additively manufactured RPD.

**FIGURE 7:**
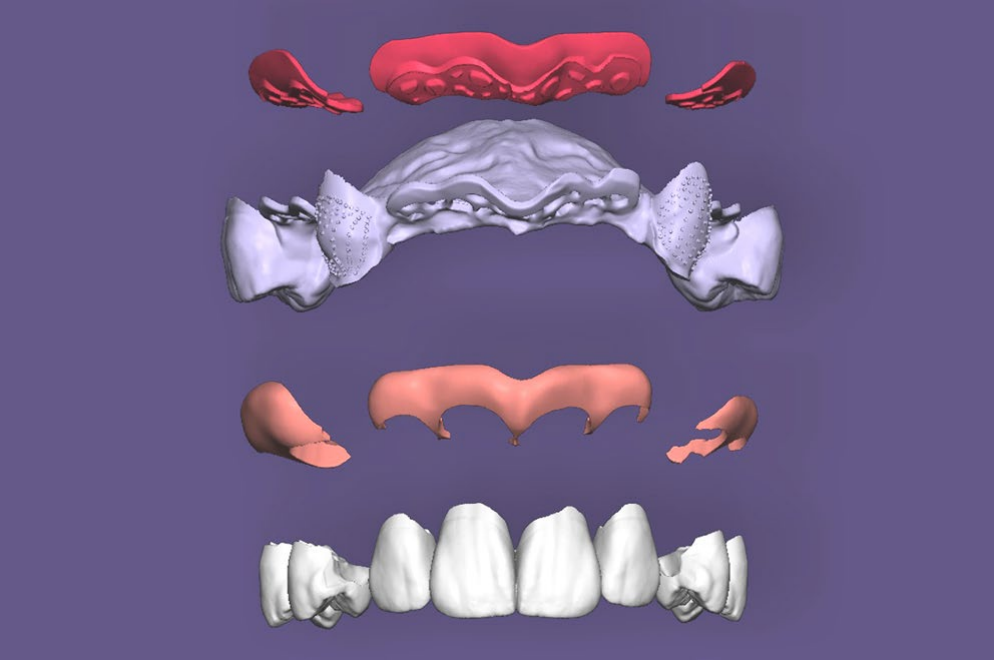
CAD (PartialCAD; Exocad) of a RPD depicting the different parts (resin base, metal framework, gingival mask, and teeth) to be 3D‐printed separately and then assembled.

Early workflows used milled or printed resin/wax patterns for casting, but current developments increasingly allow direct 3D printing of metal frameworks. Frameworks and teeth can be printed separately and combined by adding the pink resin parts (Figure 6a–c), and even hybrid printing of all metal and resin components with minimal analog steps for assembly is possible, further improving efficiency (Figure 7).

**FIGURE 8:**
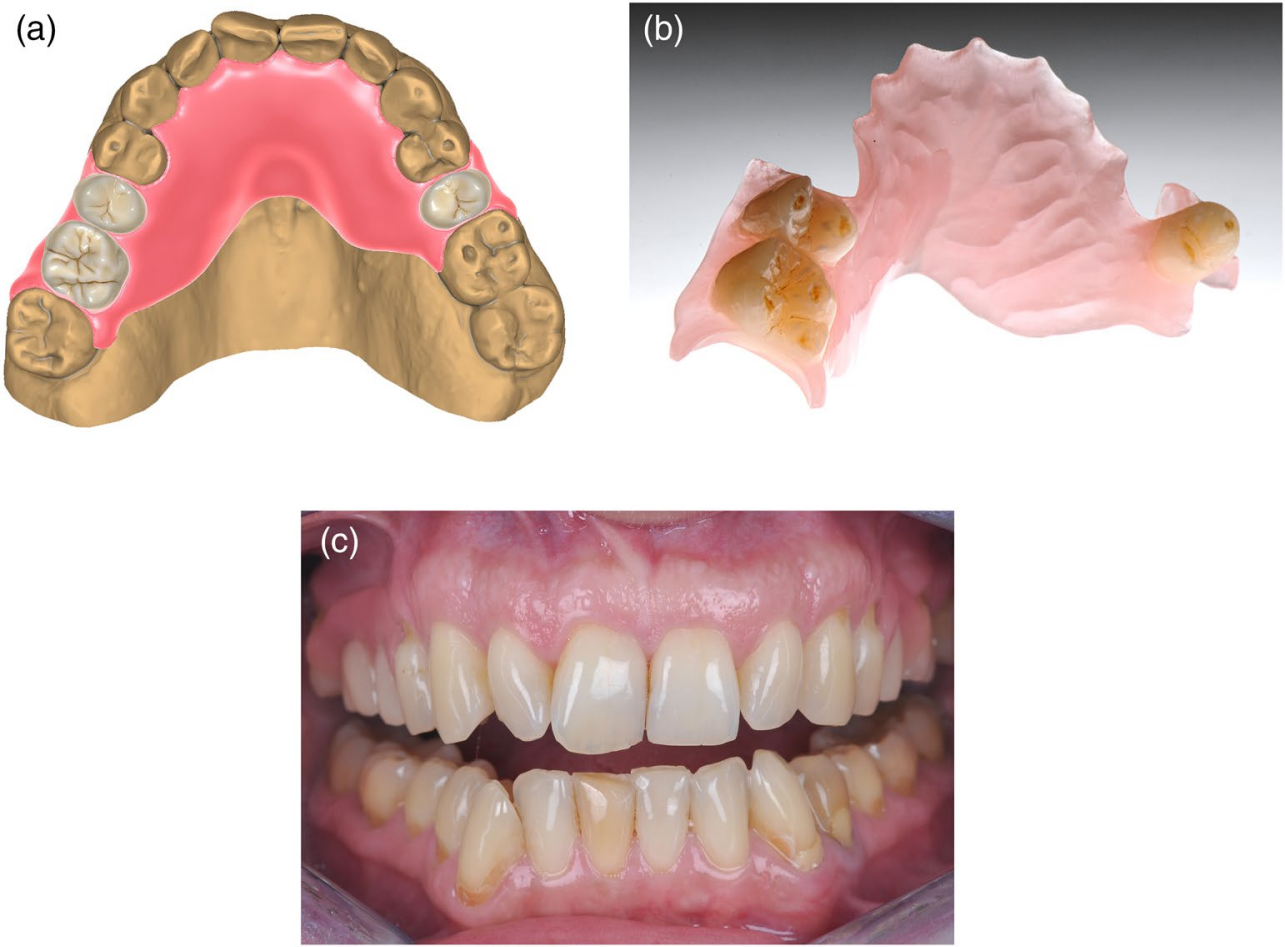
(a) CAD (DentaCAD; Exocad) of an interim RPD without metal framework. (b) Interim RPD without metal framework, with 3D‐printed teeth (Freeprint crown A3; Dentax, Ettlingen, Germany) and base (Dentax; Freeprint denture flex) firmly connected and finalized. All parts were printed with the Asiga max 3D printer (Asiga, Sydney, Australia). (c) Metal‐free 3D‐printed maxillary interim denture, discretely integrating into the dentition.

Beyond metal frameworks, digital workflows can produce esthetic, metal‐free RPDs from high‐performance polymers, offering lightweight and resource‐efficient interim or definitive options [26]. In these cases, a modified clasp design is used, creating “wings” that engage the tooth undercuts (Figure 8a–c).

**FIGURE 9:**
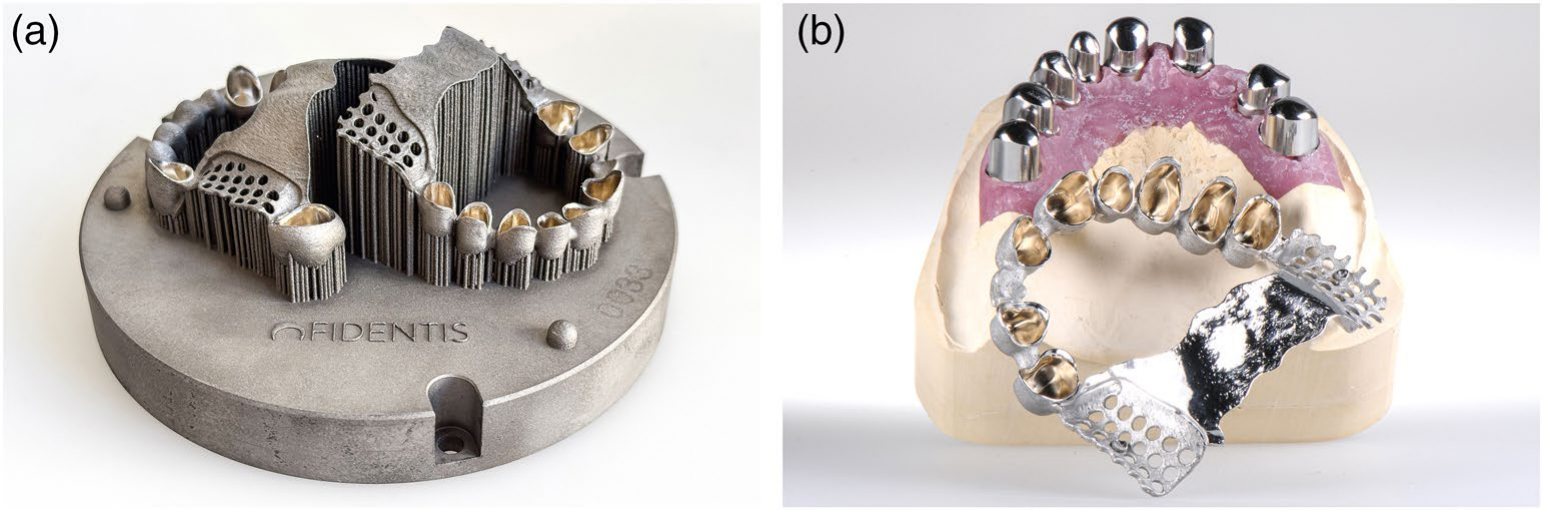
(a) Multi‐material 3D printing of double crown denture framework fabricated using DMLS, combining precious metal (Elfenbeingold G2, Hafner, Pforzheim, Germany) and non‐precious metal alloys (Starbond CoS Powder, Scheftner, Mainz, Germany) in a single build (Fidentis, Augsburg, Germany). (b) Double‐crown denture framework with a thin gold inner layer and CoCr as the main framework material (Fidentis). Primary crowns are milled from CoCr.

Another innovation is multi‐material printing for double‐crown technology, enabling one‐piece frameworks with precious and non‐precious alloys—for example, a thin gold layer on the intaglio surface of secondary crowns for improved friction (Figure 9a,b) [27].

### 2.3 3D Printing in Implant‐Retained Overdentures

**FIGURE 10:**
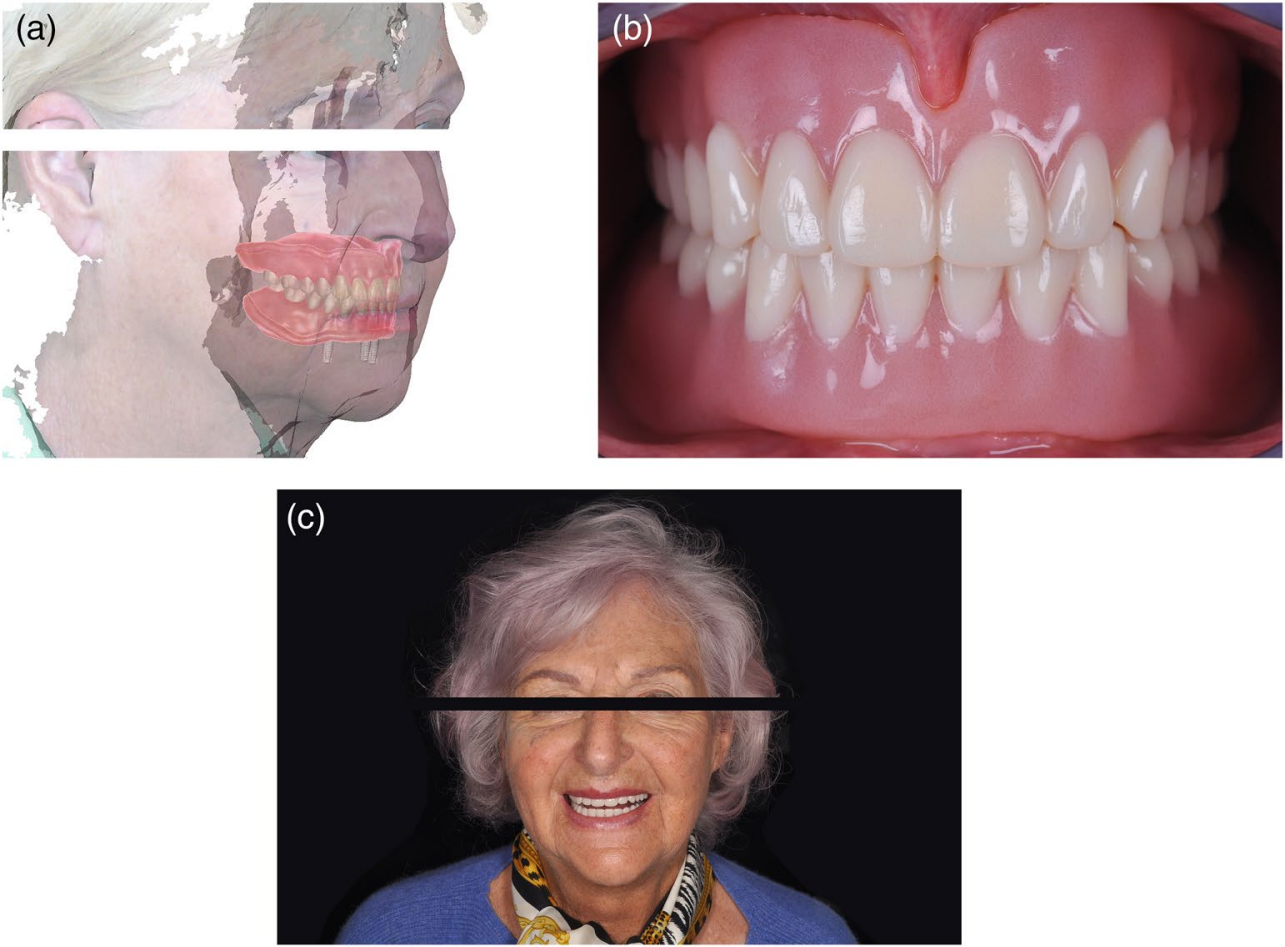
(a) Digital twin of a patient with CAD (DentalCAD; Exocad) of a 3D‐printed multi‐material maxillary complete denture and mandibular implant‐supported overdenture. (b) 3D‐printed multi‐material denture and implant‐supported overdenture in situ (TrueDent Denture, proof‐of‐concept case; Stratasys). The lower denture is supported by locator attachments (Straumann Novaloc; Straumann, Basel, Switzerland). (c) Portrait of the patient wearing the complete denture and implant overdenture.

Implant‐retained overdentures benefit from polymer 3D printing for denture bases and teeth, as well as from 3D printing of bars and frameworks. SLM and DMLS enable direct fabrication of titanium and cobalt–chromium structures, eliminating casting steps. Recent studies show that printed frameworks achieve clinically acceptable fit and precision, in some cases comparable or superior to milled alternatives [28, 29]. Figures 10a–c illustrate the fully digitized workflow for producing an implant‐supported complete denture. AM is also applied to attachment housings and custom abutments, supporting individualized treatment concepts within fully digital workflows.

### 2.4 3D Printing in Occlusal Splints

**FIGURE 11:**
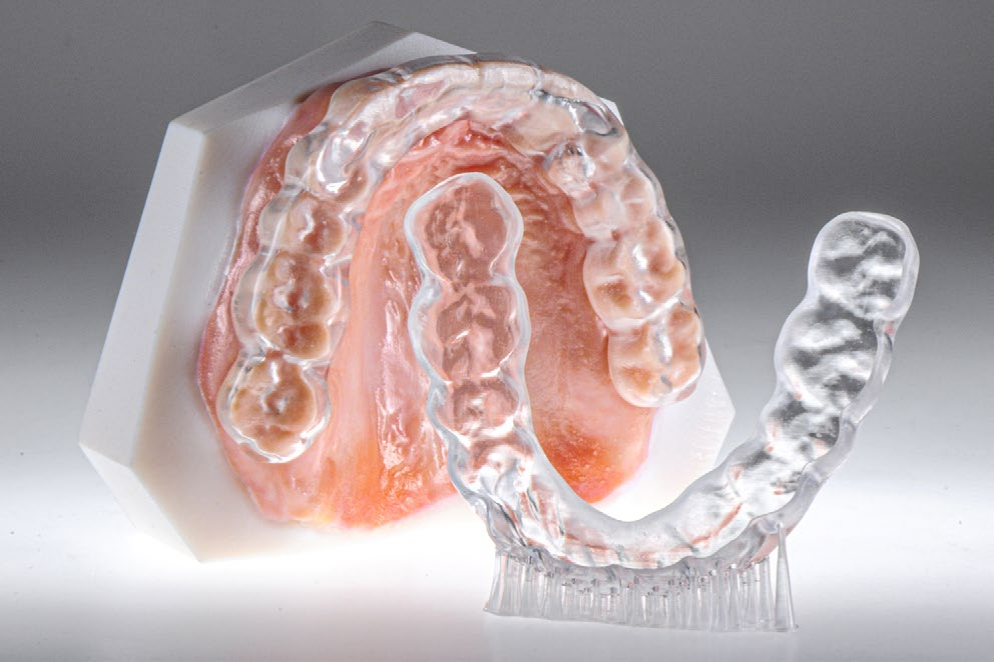
3D‐printed occlusal splint (Freeprint splintmaster taff; Detax, Ettlingen, Germany) for temporomandibular joint (TMJ) treatment, produced with a glossy finish (Asiga Max, UltraGloss; Asiga).

**FIGURE 12:**
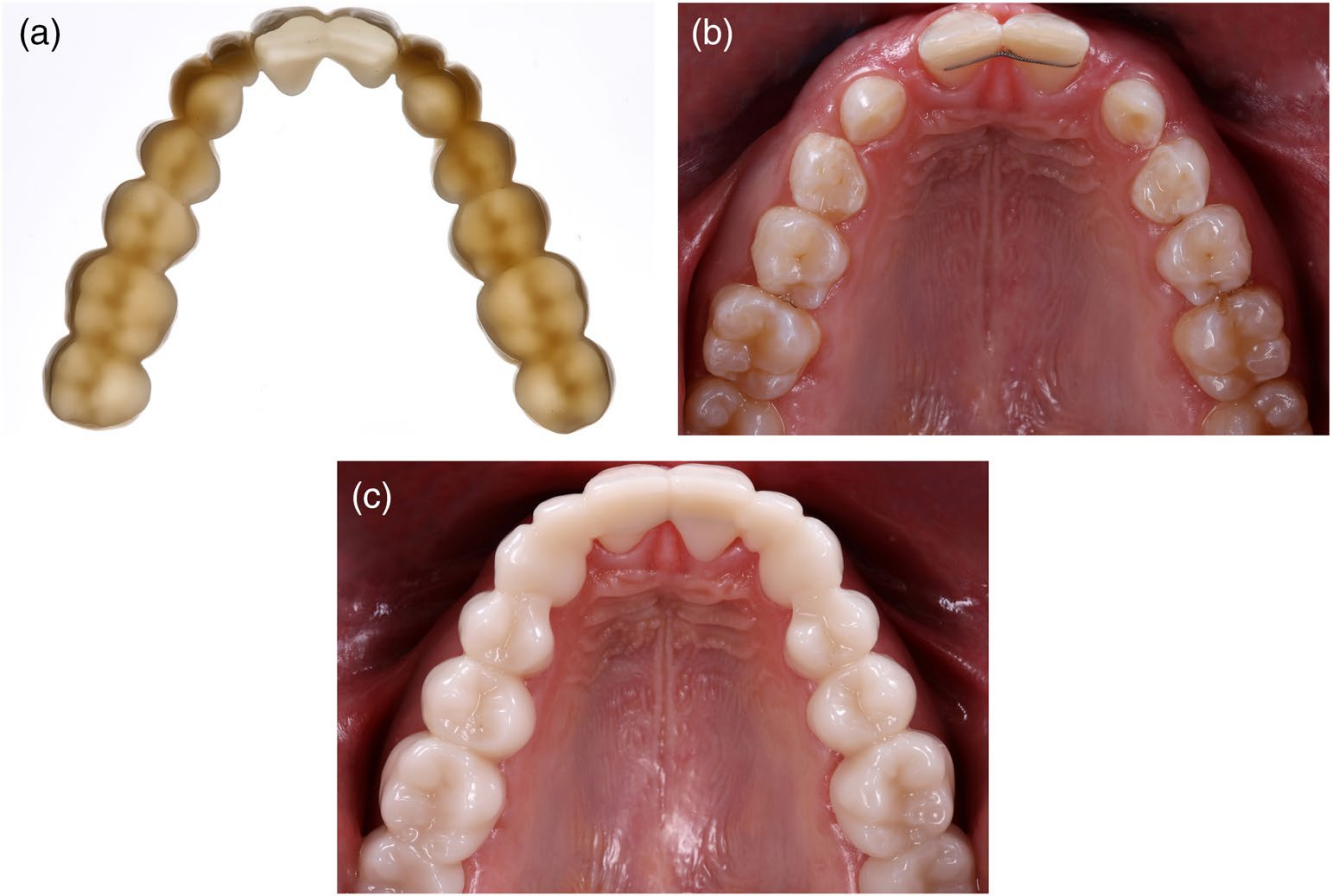
(a) Future indication for 3D printing: A splint made from tooth‐colored resin to evaluate functional and esthetic parameters before definitive restoration (“Munich Splint”). (b) Clinical situation of a patient with multiple agenesis and persistent primary teeth. (c) The splint is integrated to provide an enhanced esthetic appearance, to allow esthetic evaluation and discussion of possible modifications, and to assess an increased vertical dimension of occlusion.

Occlusal splints are among the most interesting applications of dental 3D printing. Vat‐photopolymerization enables transparent splints with high accuracy (Figure 11) [13]. Compared with vacuum‐formed or conventionally processed devices, printed splints provide precise fit, sufficient strength, and easy replacement, which is especially valuable in patients with bruxism or temporomandibular disorders [13, 16]. Production is steadily increasing, and extended applications are emerging, including orthodontic aligner therapy [11] and bimaxillary removable simulation splints in tooth‐colored resin (“Munich Splint Concept”) for esthetic and functional evaluation (Figure 12) [30, 31].

## 3 Discussion

Modern dental 3D printing enables efficient production of complex removable prostheses while maintaining consistent quality, making it a key technology for the future. Particularly in removable prosthodontics, additive manufacturing is well suited for large‐volume, patient‐specific geometries that often require multiple materials and individualized designs.

For complete dentures, clear advantages include streamlined digital workflows, reduced clinical visits, rapid duplication, and digital archiving [17, 20]. Digital trial dentures facilitate esthetic and functional verification before finalization, and systematic reviews confirm that patient‐reported outcomes in 3D‐printed complete dentures are largely comparable to those of conventional approaches [17]. In addition, the possibility of archiving digital data allows reproduction and long‐term documentation [19]. Duplicate or copy dentures also allow for straightforward fabrication not only in cases of damage or loss, but also when teeth are worn, esthetic alterations are desired, or the fit must be re‐established through relining.

However, limitations remain. 3D‐printed dentures provide acceptable adaptation discrepancies of approximately 50–150 μm, good retention, and favorable patient‐reported outcomes [17, 32, 33]. They show lower flexural strength, hardness, and bond strength than milled PMMA blanks [14, 34, 35]. Cost analyses suggest that while chairside time may be reduced, laboratory time and material costs may vary considerably [33, 36].

Process variability and lack of standardized workflows further affect outcomes [37, 38]. The lack of standardized workflows contributes to heterogeneity in clinical outcomes [17]. In addition, the bond strength between artificial teeth and 3D‐printed denture base resins is still inferior to conventional combinations [39], and accurate IOS of edentulous arches also remains challenging [6]. Long‐term concerns include fracture resistance, discoloration, wear, and microbial colonization [13, 19, 35, 40].

Future developments are promising. Multi‐material fabrication may eliminate bonding steps, improve structural integrity, and enable monolithic dentures. Material jetting technologies, such as PolyJet printing, allow multi‐material and multi‐color fabrication while avoiding sagging [41, 42] and reducing the need for support structures, thereby minimizing deformation and post‐processing‐related inaccuracies [32].

The integration of different material properties, such as color gradients or region‐specific mechanical behavior, already at the CAD stage represents a major advantage of additive manufacturing. Novel printable resins with nanoparticle reinforcement, antimicrobial activity, or resilient properties may improve durability and comfort [11, 13, 14]. Still, robust long‐term clinical studies are urgently required.

For RPDs, 3D printing has reached clinical applicability. Studies confirm acceptable accuracy and fit of below 100 μm, with SLM frameworks showing high tensile and fatigue strength that may equal or exceed those of conventionally cast alloys [23, 25, 43]. Digital workflows reduce chairside time, improve reproducibility, and support patient acceptance [21, 22]. Nevertheless, long‐term clinical and biological outcome data remain limited.

A novel approach in dental 3D printing is the multi‐material DMLS of double crown dentures, combining precious with non‐precious metal alloys. This method enables “gold‐like” friction with minimal use of only a thin inner layer of gold [27]. The largely automated approach might enable the sophisticated technology of double crown dentures to become more broadly established.

Beyond metal frameworks, digital workflows also allow fabrication of RPDs from polymers [26]. These provide esthetic benefits without visible clasps, are lightweight, and avoid metal alloys. Digital fabrication enables reproducible designs and easy refabrication, and multi‐material printing could further support efficient monolithic production. However, polymers show lower rigidity, with clasps prone to deformation or fracture, and current clinical evidence remains scarce. A modified clasp design, further mechanical validation, and long‐term studies are needed before broad adoption.

In implant overdentures, AM allows integration of printed bases, frameworks, and attachments, combining design freedom with digital efficiency. Clinical studies confirm acceptable accuracy and short‐term survival [28, 44]. Patient‐centered advantages include shorter treatment times, simplified refabrication, and improved comfort with IOS [5].

Nonetheless, process‐related factors such as porosities, residual stresses, and surface roughness require careful post‐processing [12], and long‐term data remain limited.

Occlusal splints are a widespread application. Printed devices provide precise fit, reproducibility, and rapid replacement, and patient comfort, but mechanical properties remain inferior to milled resins—with flexural strength values typically above 110 MPa for milled materials and below 100 MPa for printed alternatives [13, 16]. Aging, discoloration, and microbial colonization highlight the need for improved resins [13, 45].

Emerging concepts include multi‐material splints with rigid and resilient zones, sensor integration for bruxism monitoring, and bimaxillary splints made of tooth‐colored resin for esthetic and functional evaluation before final restoration [30, 31].

Looking ahead, 4D‐printing [46] may introduce adaptive prostheses with stimuli‐responsive materials, such as dentures that adjust to ridge resorption or splints with variable stiffness. Overall, 3D printing is progressing from extraoral toward intraoral applications and from temporary toward definitive indications. Its advantages—design freedom, efficiency, reproducibility, and reduced manual steps—are evident. Remaining challenges primarily concern material durability, long‐term biocompatibility, and standardized clinical validation. Addressing these aspects will be essential for the sustained integration of additive manufacturing into prosthetic dentistry and for realizing a true “data‐to‐denture” workflow.

## 4 Conclusion

3D‐printed complete dentures are now clinically feasible and can be produced efficiently. Digital workflows enable reproducible fabrication and rapid generation of copy dentures, offering clear clinical and economic advantages. Remaining challenges include mechanical reliability and limited long‐term clinical evidence, emphasizing the need for standardized protocols and prospective validation.

Future developments will focus on multi‐material printing, enabling monolithic dentures and hybrid polymer–metal structures. Multi‐material DMLS supports advanced applications such as double‐crown dentures with gold‐like friction.

For RPDs, workflows aiming to print all components in a single process and fuse them afterwards, as well as metal‐free alternatives, offer promising options. Implant overdentures may likewise benefit from multi‐material strategies, lighter lattice frameworks, and AI‐driven design.

Multi‐material concepts and novel applications, such as esthetic tooth‐colored bimaxillary splints or orthodontic aligner therapy, highlight the expanding clinical potential and near‐future applications of printed devices.

## Conflicts of Interest

The authors declare no conflicts of interest.

## Data Availability

The data that support the findings of this study are available on request from the corresponding author. The data are not publicly available due to privacy or ethical restrictions.
